# Editorial: Neuroimaging of Affective Empathy and Emotional Communication

**DOI:** 10.3389/fneur.2018.00875

**Published:** 2018-10-26

**Authors:** Argye E. Hillis

**Affiliations:** Johns Hopkins University School of Medicine, Baltimore, MD, United States

**Keywords:** emotion, functional neuroimaging, prosody, empathy, lesion-deficit brain mapping

This e-book brings together studies of the neural networks underlying affective empathy and emotional communication, from investigators from eight countries. The studies use a variety of methodologies, including EEG, task-related fMRI, resting state fMRI, PET, measures of cortical structure, parcel-based lesion symptom mapping, voxel based morphometry (VBM), facial electromyography, and real-time fMRI feedback. The investigations included a variety of populations, including healthy participants and people with hearing loss, autism, frontotemporal dementia (FTD), Alzheimer's disease (AD), primary progressive aphasia (PPA), progressive supranuclear palsy (PSP), corticobasal syndrome (CBS), and focal brain lesions. Although results do not converge on a single neuroanatomical or functional model of affective empathy or communication of emotions, they provide new insights into how we express, recognize, and share the emotions of other people.

Lorenzetti et al. studied emotion regulation in eight healthy controls using real-time fMRI neurofeedback (rtfMRI-NFB), as a proof of concept that this procedure might be useful in modulating complex emotions in people with anxiety, stress, or impaired empathy. The rtfMRI-NFB software provided a virtual environment to induce tenderness and anguish, using brain-computer interface and music. The procedure provided a robust method for both real-time measurement of the neural correlates of tenderness and anguish and voluntary modulation of these emotions. During tenderness, participants recruited the septo-hypothalamic area, medial frontal cortex, temporal pole, and precuneus. During anguish, participants recruited the amygdala, dorsolateral prefrontal, and additional regions associated with negative affect.

Zinchenko et al. reported data from two EEG experiments of 21 participants with hearing loss and 21 age-matched healthy controls, who observed multimodal video clips with facial expressions matched or mismatched with corresponding vocalizations. Participants categorized emotions (emotional conflict) of the clips. Negative stimuli modulated behavioral conflict processing in the normal hearing, but not in the hearing loss group. Yet, the amplitude difference in N100 responses between congruent and incongruent stimuli was larger in negative relative to neutral conditions in both groups across tasks. In the emotional conflict task, the hearing loss group performed at chance. They conclude that hearing loss affects processing of emotional acoustic cues and alters the behavioral benefits of emotional stimuli on cognitive and emotional control, despite preservation of early neural responses.

Pereira et al. report differences in cortical structure and functional connectivity in the default mode network (DMN) in rsfMRI in 22 participants with high-functioning autism (ASD) compared to 29 healthy controls. ASD patients had decreased gray matter volume and cortical thickness in cingulate, temporal lobes, and amygdala. Participants with ASD had reduced connectivity between the posterior cingulate cortex and areas of the executive control component of the DMN and higher connectivity between the anteromedial prefrontal cortex and areas of the sensorimotor component of the DMN. Decreased cortical thickness in right inferior frontal lobe correlated with poorer social functioning.

Healy and Grossman reviewed evidence from functional imaging in healthy participants and from behavioral and structural imaging studies in patients with bvFTD to identify shared and separate anatomical substrates of cognitive perspective-taking (ability to infer another's thoughts or beliefs) and affective perspective-taking (ability to infer another's emotions). They report that both types of perspective-taking engage temporoparietal junction, precuneus, and temporal poles, while only affective perspective-taking engages limbic system regions and basal ganglia. Furthermore, cognitive perspective-taking engages dorsomedial and dorsolateral prefrontal cortex, while affective perspective-taking engages ventromedial prefrontal cortex.

Pressman et al. examined the neural regions critical for shared conversational laughter in 75 participants with neurodegenerative disease, including AD, bvFTD, PPA, CBS, and PSP. In tapes of brief unrehearsed conversation with a partner, laughter was manually labeled and the timing of that laughter relative to the partner's laughter was identified. A voxel-based morphometry analysis of abnormal timing of laughter revealed the role of left precuneus and right fusiform gyrus.

Carr and Mendez carried out a meta-analysis of studies of affective empathy in bvFTD, who typically have atrophy of medial prefrontal, insula, and anterior temporal cortex. They found that people with bvFTD showed only a modest impairment in affective compared to controls across tasks. The most marked impairment was found in empathic concern.

Hua et al. reported evidence that empathy in bvFTD is undermined by enhanced positive emotional reactivity. They used facial electromyography of 26 participants with bvFTD and 25 healthy controls, as they identified emotions displayed in photographs of positive, negative, and neutral emotional faces. Participants with bvFTD showed impaired emotion recognition and greater reactivity of Zygomaticus major (ZM) (which is active during positive emotional reactions like smiling), compared to controls. Higher ZM reactivity was associated with worse negative emotion recognition. VBM revealed that smaller volume in the thalamus, midcingulate cortex, posterior insula, anterior temporal pole, amygdala, precentral gyrus, and inferior frontal gyrus was associated with greater ZM reactivity in bvFTD.

Beadle et al. identified a critical role of ventromedial prefrontal cortex in helping others who are suffering, by inducing empathy in eight participants with focal damage to the vmPFC and healthy controls, and measuring in real time their emotional responses and empathetic behavior. Those with damage to the vmPFC gave less money than healthy participants to a confederate who was suffering (a confederate).

Patel et al. found that listener ratings of prosody in 41 acute ischemic RH stroke patients positively correlated with four acoustic measures. Reductions in each of these four “prosody acoustic measures” was predicted by lesion load in pars opercularis, supramarginal gyrus, or associated white matter tracts (and not control regions).

Finally, Sidtis and Sidtis reviewed evidence from fMRI and PET studies of healthy participants and lesion-deficit association studies, of the neural bases of formulaic language, such as expletives and idioms that convey emotions. Evidence suggests that a right hemisphere-subcortical network modulates formulaic language.

Together, these studies provide evidence for a complex right-dominant network, including (but not limited to) ventromedial prefrontal cortex, amygdala, inferior frontal gyrus, insula, and temporal pole that mediates expression and recognition of emotion as well as emotional empathy. Each study reveals unique, novel insights about this complex network and stimulates future research in this field.

## Author contributions

The author confirms being the sole contributor of this work and has approved it for publication.

### Conflict of interest statement

The author declares that the research was conducted in the absence of any commercial or financial relationships that could be construed as a potential conflict of interest.

